# Impact of Benign Prostatic Hyperplasia Pharmacological Treatment on Transrectal Prostate Biopsy Adverse Effects

**DOI:** 10.1155/2014/271304

**Published:** 2014-04-28

**Authors:** Marina Zamuner, Ciro Eduardo Falcone, Arnaldo Amstalden Neto, Tomás Bernardo Costa Moretti, Luis Alberto Magna, Fernandes Denardi, Leonardo Oliveira Reis

**Affiliations:** ^1^Urology Division, Faculty of Medicine, Pontifical Catholic University of Campinas (PUC-Campinas), 13083-970 Campinas, SP, Brazil; ^2^Dr. Mario Gatti County Hospital, 13083-970 Campinas, SP, Brazil; ^3^Urology Division, Department of Surgery, University of Campinas (UNICAMP), 13083-970 Campinas, SP, Brazil; ^4^Department of Genetics, University of Campinas (UNICAMP), 13083-970 Campinas, SP, Brazil; ^5^Department of Urology, School of Medical Sciences, University of Campinas (UNICAMP), 13083-970 Campinas, SP, Brazil

## Abstract

*Background*. Benign prostatic hyperplasia (BPH) pharmacological treatment may promote a decrease in prostate vascularization and bladder neck relaxation with theoretical improvement in prostate biopsy morbidity, though never explored in the literature. *Methods*. Among 242 consecutive unselected patients who underwent prostate biopsy, after excluding those with history of prostate biopsy/surgery or using medications not for BPH, we studied 190 patients. On the 15th day after procedure patients were questioned about symptoms lasting over a week and classified according to pharmacological BPH treatment. *Results*. Thirty-three patients (17%) were using alpha-blocker exclusively, five (3%) 5-alpha-reductase inhibitor exclusively, twelve (6%) patients used both medications, and 140 (74%) patients used none. There was no difference in regard to age among groups (*P* = 0.5). Postbiopsy adverse effects occurred as follows: hematuria 96 (50%), hematospermia 53 (28%), hematochezia 22 (12%), urethrorrhagia 19 (10%), fever 5 (3%), and pain 20 (10%). There was a significant negative correlation between postbiopsy hematuria and BPH pharmacological treatment with stronger correlation for combined use of 5-alpha-reductase inhibitor and alpha-blocker over 6 months (*P* = 0.0027). *Conclusion*. BPH pharmacological treatment, mainly combined for at least 6 months seems to protect against prostate biopsy adverse effects. Future studies are necessary to confirm our novel results.

## 1. Introduction

Biopsy of the prostate to diagnose or exclude cancer is performed nearly one million times annually in the USA [1]. The main tests used for early prostate cancer detection are (1) digital rectum examination (DRE) and (2) prostate-specific antigen (PSA); the first is a subjective and examiner-dependent test, and the second is not always correlated with prostate malignancy [2–4], so the method of choice for a conclusive diagnosis is transrectal ultrasound guided prostate biopsy, with a rising concern regarding its collateral effects and complications nowadays [5].

Prostate biopsy may be associated with a significant rate of adverse effects in over 80% of the patients [6], such as pain, lower urinary tract symptoms, urinary retention, erectile dysfunction, and more frequently bleeding and infection [7].

Pharmacological treatment for benign prostate hyperplasia (BPH) classically comprises two drug classes: 5-alpha-reductase inhibitors and alpha-blockers that may promote a decrease in prostate tissue vascularization [8–10] and bladder neck and prostate relaxation [11], which might lead to a theoretical improvement in prostate biopsy morbidity and complications, although never tested before in the literature.

This study aims to evaluate prostate transrectal biopsy adverse effects in patients after pharmacological BPH treatment compared to patients not under BPH treatment.

## 2. Methods

### 2.1. Population

This study was performed in accordance with institutional ethical guidelines, based on good clinical practice. We retrospectively studied a prospectively collected database of 242 unselected and consecutive male adults who underwent transrectal ultrasound guided prostate biopsy in a general urologic clinic from a community hospital in 2012.

### 2.2. Biopsy Indications

All patients with a PSA value greater than 4.0 ng/mL or suspicious DRE were considered eligible for prostate biopsy [12].

### 2.3. Exclusion Criteria

Patients with previous history of prostate biopsy or prostatic surgery, those taking additional medications, and those who presented adverse effects due to BPH pharmacological treatment or undertreatment for less than 6 months were excluded from analysis (*n* = 52).

### 2.4. Prostate Biopsy

All patients underwent 12-core transrectal ultrasound guided prostate biopsy performed by a single urologist, using a side-notch cutting biopsy needle (18 G, 19-mm stroke length). The biopsies were performed following rectal enema and local anesthesia 10 mL of lidocaine 2% in the neurovascular bundle bilaterally (5 mL each side) via a 6.5 Hz probe ultrasound (Logiq 100; GE Medical Systems, Milwaukee, WI).

The primary peripheral zone biopsy localizations were apical, middle, and base, being 2 cores each zone: one paramedian and another lateral [13].When there was a nodule on digital rectal prostate examination or a hypoechoic area was detected through ultrasound, 02 additional biopsy cores were taken from these sites [14].

### 2.5. Previous Pharmacological Treatment for BPH

Patients were classified according to the chronic use of 5-alpha-reductase inhibitor (finasteride 5 mg/day or dutasteride 0.5 mg/day) and/or alpha-blockers (doxazosin 4 mg/day or tamsulosin 0.4 mg/day) for at least 6 months.

### 2.6. Analysis of Adverse Effects

According to the local institutional surveillance protocol for adverse effects, on the 15th day after the biopsy, patients were interviewed and questioned regarding possible biopsy adverse effects lasting more than one week after the procedure such as hematuria, hematospermia, hematochezia, and urethrorrhagia. Additionally, fever after procedure was defined as elevation of body temperature above 37.5°C (99.5°F), at least one episode. Possible answers were Yes or No.

A: Post procedure pain was graded based on a visual analog scale (VAS; 0 = none to 10 = worst pain) and those patients referring to VAS ≥ 5 24 h after the procedure were classified as clinically significant pain.

### 2.7. Statistics

Variables were expressed as mean ± standard deviation (range). Student *t*- and Fisher tests were used when indicated and two-sided  *P* < 0.05 was considered significant.

## 3. Results

Among patients prospectively analyzed (*n* = 190), thirty-three patients (17%) were using alpha-blocker exclusively, mean age 62 ± 8 (39–89) years; five (3%) patients used 5-alpha-reductase inhibitor exclusively, mean age 64 ± 9 (45–72); twelve (6%) patients used both medications, mean age 68 ± 6 (58–79) years; and 140 (74%) patients used none, mean age 63 ± 9 (43–87) years. There was no difference in regard to age among groups (*P* = 0.5).

Postbiopsy adverse effects occurred as follows: hematuria 96 (50%), hematospermia 53 (28%), hematochezia 22 (12%), and urethrorrhagia 19 (10%). Fever occurred in 5 (3%) and significant pain in 20 (10%). No patient presented severe adverse effect such as sepsis or fever for more than 72 h and there was no hospitalization need in the reported series.

There was a significant negative correlation between postbiopsy hematuria and previous use of medications for BPH (*P* = 0.01), being the stronger correlation for those under combined use of 5-alpha-reductase inhibitor and alpha-blocker for over 6 months (*P* = 0.0027) (Table 1).

## 4. Discussion

Our results are hypothesis generating and show for the first time in the literature that BPH pharmacological treatment and mainly the combined therapy of 5-alpha-reductase inhibitors and alpha-blockers for at least 6 months may protect against hematuria after prostate biopsy.

The pharmacological treatment of BPH, mainly the 5-alpha-reductase inhibitors, may reduce the prostate microvessel density, which could, theoretically, decrease the rate of hemorrhagic symptoms after prostate biopsy [8, 15].

Alpha-blockers acting as bladder neck and prostate relaxants can also potentially impact prostate biopsy adverse effects. Specifically regarding hemorrhagic adverse effects that we showed to be the most frequent, blockade of alpha-adrenergic receptors reduces the sympathetic tone of blood vessels resulting in decreasing vascular resistance, reducing blood pressure, and eventually minimizing local hemorrhagic adverse effects.

Additionally, Keledjian et al. studying human prostate showed incremental apoptosis with decreased cell proliferation, microvessel density, vascular endothelial growth factor (VEGF), and prostate specific antigen (PSA) immunoreactivities in patients under alpha-blockers [10]. However, the present study is the very first to consider these supposed mechanisms in the prostate biopsy scenario, warranting further analyses.

Prostate biopsy is generally well tolerated, with a low risk of major complications such as infection and sepsis. Minor complications also called adverse effects such as pain and bleeding are frequent and represented by hematuria, hematospermia, and hematochezia [16, 17]. Our results showed an occurrence of fever in only 3% of the patients and hemorrhagic adverse effects in over 80%.

Prostate biopsy causes important pain, discomfort, and anxiety in most patients, mainly during the procedure, but there are scarce data dealing with pain in the days after the biopsy [18]. Our results show that only 10% of all patients had significant pain on subsequent days.

Kravchick et al. showed that 6 weeks of dutasteride treatment might reduce prostate tissue vascularity in the periurethral area proximal to the verumontanum [8]. Thus it was logical to apply this condition into a surgical situation, as did a few authors by analyzing the postoperative bleeding in patients using 5-alpha-reductase inhibitors. They showed that pretreatment with dutasteride for 4 to 6 weeks before transurethral resection of the prostate reduces the surgical bleeding considerably [19, 20].

Other authors focused on the postoperative complications. Hahn et al. showed no significant reductions in postoperative bleeding among patients taking 5-alpha-reductase for 2 weeks before transurethral resection of the prostate [21]. In a similar study, Arratia-Maqueo et al. also showed no significant reductions in postoperative bleeding among patients taking the same medicine for a longer time period of 4 weeks [22].

Whereas the rational for 5-alpha-reductase inhibitors impacting adverse effects has been explored in the context of prostate transurethral resection (TUR), alpha-blockers acting as bladder neck and prostate relaxants can also potentially impact prostate manipulation adverse effects.

While controversial, the effect of 5-alpha-reductase inhibitors was never proposed in the setting of prostate biopsy and the impact of alpha-blockers was underexplored even in the TUR scenario. The current study is the first in the literature to measure the impact of 5-alpha-reductase inhibitors and alpha-blockers on prostate biopsy adverse effects.

Although based on a prospectively collected database with unprecedented original rational, the study has some limitations.

First, a relatively small number of patients were compared after grouping, impeding subgroup analyses of different drugs in the same class (i.e., finasteride versus dutasteride and doxazosin versus tamsulosin), even though of minor importance. Second, the grade of low urinary tract symptoms (LUTS) and prostate volume were not measured in this series, factors that certainly determined pharmacological treatment indication.

Additionally, patients under medications for less than 6 months were excluded and adverse effects were graded as lasting over a week or not, an arbitrary criterion, although objective and easy to measure.

Last but not least, this study design although important as hypothesis generating cannot precisely distinguish if differences observed are regarding medicines utilized and their potential effects or regarding BPH/LUTS that culminated with BPH treatment.

Future studies including larger series of patients with a wide range of LUTS and prostate volumes, with randomized and placebo controlled design, assessing the impact of specific monotherapy, including additional information and with different BPH pharmacological treatment intervals (i.e., one, three, six, twelve, and over months) and refining the adverse effects grading criteria, are warranted to expand knowledge regarding this underexplored issue.

## 5. Conclusions

In this preliminary original and hypothesis generating study, BPH pharmacological treatment, mainly combined use of 5-alpha-reductase inhibitors and alpha-blockers for at least 6 months, might protect against hematuria after prostate biopsy. Future larger studies must be conducted to confirm our innovative results.

## Figures and Tables

**Table 1:** Correlation between prostate biopsy adverse effects and pharmacological treatment for benign prostatic hyperplasia.

Adverse effects*	Control *n* = 140	Group 1 *n* = 33	*P*	Group 2 *n* = 5	*P*	Group 3 *n* = 12	*P*
Hematuria	82 (59%)	12 (36%)	** 0.01**	1 (20%)	**0.02**	1 (8%)	**0.003**
Hematospermia	42 (30%)	6 (18%)	0.2	3 (60%)	0.1	2 (17%)	0.5
Hematochezia	16 (11%)	4 (12%)	1	1 (20%)	0.5	1 (8%)	1
Urethrorrhagia	12 (9%)	6 (18%)	0.1	0 (zero)	1	1 (8%)	1
Fever	4 (3%)	1 (3%)	1	0 (zero)	1	0 (zero)	1
Pain**	16 (11%)	2 (6%)	0.5	1 (20%)	0.5	1 (8%)	1

Control: patients not using any medication (control/reference). Group 1: patients using alpha-blockers, exclusively. Group 2: patients using 5-alpha-reductase, exclusively. Group 3: patients using both medications. *P* value ≤0.05 was considered significant (bold).*Lasting more than one week after the procedure. **Visual analog scale (VAS) ≥5, 24 h after the procedure.
